# Synthesis and Study of Shape-Memory Polymers Selectively Induced by Near-Infrared Lights via In Situ Copolymerization

**DOI:** 10.3390/polym9050181

**Published:** 2017-05-20

**Authors:** Tianyu Fang, Liang Fang, Shunping Chen, Lingyu Li, Hengming Huang, Chunhua Lu, Zhongzi Xu

**Affiliations:** 1 State Key Laboratory of Materials-Oriented Chemical Engineering, College of Materials Science and Engineering, Nanjing Tech University, Nanjing 210009, China; fangtianyu0105@163.com (T.F.); chensp@njtech.edu.cn (S.C.); lilingyu1216@163.com (L.L.); huanghengming@njtech.edu.cn (H.H.); zzxu@njtech.edu.cn (Z.X.); 2 Jiangsu Collaborative Innovation Center for Advanced Inorganic Function Composites, Nanjing Tech University, Nanjing 210009, China; 3 Jiangsu National Synergetic Innovation Center for Advanced Materials (SICAM), Nanjing Tech University, Nanjing 210009, China

**Keywords:** shape-memory polymer, photo-responsive, rare earth organic complexes, near-infrared light

## Abstract

Shape-memory polymers (SMPs) selectively induced by near-infrared lights of 980 or 808 nm were synthesized via free radical copolymerization. Methyl methacrylate (MMA) monomer, ethylene glycol dimethylacrylate (EGDMA) as a cross-linker, and organic complexes of Yb(TTA)_2_AAPhen or Nd(TTA)_2_AAPhen containing a reactive ligand of acrylic acid (AA) were copolymerized in situ. The dispersion of the organic complexes in the copolymer matrix was highly improved, while the transparency of the copolymers was negligibly influenced in comparison with the pristine cross-linked PMMA. In addition, the thermal resistance of the copolymers was enhanced with the complex loading, while their glass transition temperature, cross-linking level, and mechanical properties were to some extent reduced. Yb(TTA)_2_AAPhen and Nd(TTA)_2_AAPhen provided the prepared copolymers with selective photothermal effects and shape-memory functions for 980 and 808 nm NIR lights, respectively. Finally, smart optical devices which exhibited localized transparency or diffraction evolution procedures were demonstrated based on the prepared copolymers, owing to the combination of good transparency and selective light wavelength responsivity.

## 1. Introduction

Remote-controlled actuating of polymeric materials to drive their own movements or to manipulate other substances is one rapidly developing area in advanced intelligent materials [1,2,3,4,5]. In comparison with magnetic [2] and electrical fields [5,6] as another two widely reported non-contact stimuli, light is a precise, localized, and remote energy source [1], while its intensity [7], wavelength [8,9,10,11], irradiation position [10], irradiation frequency [11], and polarization [12,13,14] can all be adjusted. Light-responsive polymers thus have attracted increasing interest driven by their applications in actuators [15], self-walking or self-swimming devices [16], microfluidic chips [17,18], optical oscillating generators [19,20], and medical devices [21].

Shape-memory polymer (SMP) is a kind of polymer that has the ability to recover from a temporary shape to its permanent one. Superior to liquid-crystalline polymers [12,13,14,15], hydrogels [22,23,24,25], and carbon nanotube (CNT) or graphene based polymer bilayer films [26,27,28], SMP provides the possibility to define its initial and final shapes in an on-demand manner [4,29,30,31]. A convenient approach to prepare photo-responsive SMPs is to mix thermally-induced SMPs with photothermal fillers, which absorb light energy and transfer it into heat [3]. When the increased temperature resulting from the internally generated heat is above the switching temperatures (*T*_sw_s) of such SMP composites (SMPCs), shape recoveries are triggered. Various substances presenting photothermal effect have been explored as the functional fillers for SMPCs, especially carbon nanomaterials [7,32] and noble-metal nanostructures [9,10]. The response wavelength has been reported to be located in UV [33], visible [9,10], or infrared light regions [7,34]. The assembly of such selectively photo-responsive SMPCs enables the multi-shape variation upon sequenced light irradiations. The most widely reported deformation manner is the sequential deployment of conjoint responsive SMPCs with temporary zigzag shapes [11,35,36]. In addition, selectively photothermal fillers act as the actuating hinge to trigger the folding of different areas of a SMP film upon switching the irradiation order [37]. To date, light wavelengths which have been collaboratively used in not only SMPs but other light-induced polymers include UV–Vis [38], UV–NIR [39], Vis–Vis [37], Vis–NIR [18,26], and radiofrequency (RF)-RF [36]. Recently, our group reported a new photothermal filler system based on rare earth (RE) organic complexes presenting selective photothermal functions to two NIR lights which are less absorbed by human tissues [11]. The physical mixing of the prepared Yb(TTA)_3_Phen and Nd(TTA)_3_Phen powders in poly[ethylene-ran-(vinyl acetate)] (EVA) offered the shape recovery upon a NIR-NIR irradiation of 980 and 808 nm, respectively.

SMPs can be used to create a series of deformable, programmable, and shape-memory optical devices [40,41,42]. Via creating temporal microstructures on SMPs, the optical properties at both the global and local levels, especially the transparency, can be switched [40]. Furthermore, Kim et al. reported a flexible electrode with high transparency, low sheet resistance, and shape-memory capability [41]. If photo-responsive SMPCs are used in the field of smart optical devices, it is of high significance to maintain the transparency. Therefore, there is a need to improve the dispersion of functional fillers in the SMP matrix. As far as the aforementioned RE organic complexes are concerned, ligands with reactive groups have been applied to allow for in situ reactions with elastomers [43,44] and hydrogels [45]. For example, Zhang and coworkers created a gadolinium organic complex with double bonds and introduced it into natural rubber during in situ vulcanization [43]. More recently, Fan et al. [45] developed a europium organic complex also containing double bonds and copolymerized it with vinyl acetate monomers to prepare a novel luminous hydrogel. Obviously, in comparison with physical mixing, the in situ reaction as a chemical approach provides a potential path to improve the dispersion and compatibility of RE organic complexes as photothermal fillers in light-induced SMPs.

Here, acrylic acid (AA) with double bonds was used to prepare reactive Yb and Nd organic complexes. Selectively responsive SMPs to NIR lights were synthesized subsequently among methyl methacrylate (MMA) monomer, ethylene glycol dimethylacrylate (EGDMA), and the reactive organic complexes via in situ copolymerization. The structures and photothermal properties of the functional and reactive complexes were studied, while the properties and the light-induced shape-memory performance of the prepared SMPs were evaluated. Finally, we created temporal structures at the microscale on those SMPs and investigated their feasibility in the field of smart optical devices.

## 2. Materials and Methods

### 2.1. Materials

The α-thenoyltrifluoroacetone (HTTA, 222.18, 98%) and 1,10-phenanthroline (Phen, 180.21, 99%) were purchased from Sinopharm Chemical Reagent Company (Shanghai, China). YbCl_3_·6H_2_O (387.50, 99.99%) and NdCl_3_·6H_2_O (358.74, 99.99%) were provided by Funing Rare Earth Industrial Company (Funing, China). Acrylic acid (AA, 72.06, 98%), methyl methacrylate (MMA, 100.12, 98%), ethylene glycol dimethylacrylate (EGDMA, 198.22, 98%), 2,2′-azobis(2-methylpropionitrile) (AIBN, 164.21, 99.5%), and *N*,*N*-dimethylformamide (DMF, 73.09, 99.5%) were obtained from Aladdin, Shanghai, China. All chemicals were used without further purification.

### 2.2. Synthesis of Yb/Nd(TTA)_2_AAPhen Complexes

YbCl_3_·6H_2_O (10 mmol), NdCl_3_·6H_2_O (10 mmol), HTTA (20 mmol), AA (10 mmol) and Phen (10 mmol) were respectively dissolved in 30 mL ethanol. The ethanol solution of YbCl_3_·6H_2_O or NdCl_3_·6H_2_O was first poured into a three-necked flask at 60 °C in a magnetic stirred water bath. The ethanol solutions of ligands were subsequently added dropwise. The HTTA and AA solutions were added together before the Phen solution. The pH value of the mixture was adjusted to 6–7 using 1 mol·L^−1^ sodium hydroxide ethanol solution, before the reaction of 6 h. Subsequently, the precipitation was collected using centrifugation (10,000 rpm), before washing with water and ethanol for three times each and drying in a vacuum oven at 60 °C for 12 h.

### 2.3. In Situ Copolymerization of PMMA-Based Copolymers

Yb(TTA)_2_AAPhen (0, 0.25, or 0.5 g) or Nd(TTA)_2_AAPhen (0, 0.25, or 0.5 g) were first dissolved in DMF (4 g) and sonicated for 30 min. MMA monomer (5 g), EGDMA (0.25 g, 5 phr, parts of product per hundred parts of MMA) and AIBN (0.03 g, 0.6 phr) were subsequently added to the solution. The whole solution was prepolymerized in a glass vial (20 mL) at 65 °C for 30 min. Subsequently, the prepolymer was injected into a home-made glass container (*L* × *W* × *D* = 70 mm × 20 mm × 1 mm), which was placed in an oven at 65 °C for further copolymerization. After 12 h, the materials were taken out and then heated at 65 °C for 24 h in a vacuum oven. Three different powder loadings (0, 5, and 10 phr) were used and the samples were named as cPMMA, PMMA–Yb5, PMMA–Yb10, PMMA–Nd5, and PMMA–Nd10.

### 2.4. Creation of Temporary Microstructures

**Scheme 1:**
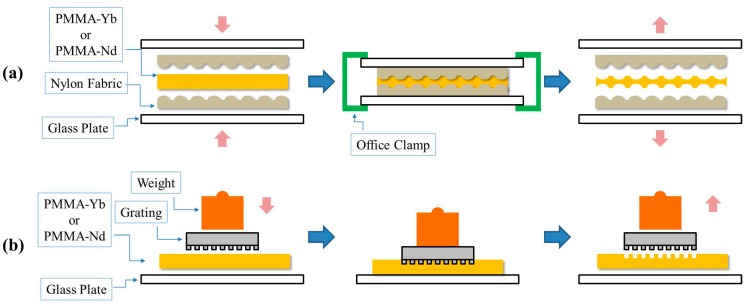
Creation procedures of temporal structures at microscale on PMMA–Yb10 and PMMA–Nd10 surfaces using (**a**) Nylon fabrics and (**b**) glass grating.

Two pieces of Nylon fabrics were pressed against both sides of PMMA-Yb10 or PMMA-Nd10, and the sandwich-structured materials were placed between two glass plates and fixed using office clamps. The materials were heated to 150 °C in an oven for 10 min and cooled down gradually to room temperature without manual intervention. The specimens with temporary surface structures were obtained by removing the clamps, glass plates, and Nylon fabrics (Scheme 1a). In addition, a piece of PMMA–Yb10 or PMMA–Nd10 specimen was placed onto a hot plate at 150 °C for 5 min. A glass grating (Deli Laser Solution Co. Ltd., Jiangyin, China) with the grating parameter of 3.3 μm was placed carefully onto the specimen and compressed using a weight of 2 kg. After 10 min, the material was cooled down to room temperature gradually (Scheme 1b) before the weight and grating were removed.

### 2.5. Characterizations

Fourier Transform Infrared Spectroscopy: Fourier transform infrared spectroscopy (FT–IR, Vector 22, Bruker, Billerica, MA, USA) was used to determine the chemical structures of the prepared Yb(TTA)_2_AAPhen and Nd(TTA)_2_AAPhen as well as the raw materials of AA, HTTA and Phen. The samples were ground with pottassium bromide (KBr) together and pressed into a testing tablet.

Reflectance and Transmittance Measurements: The reflectance spectra of Yb(TTA)_2_AAPhen and Nd(TTA)_2_AAPhen as well as the transmittance spectra of the copolymers were measured via a UV–vis–NIR spectrophotometer (UV-3101PC, Shimadzu Corp., Kyoto, Japan), using BaSO_4_ and air as the references, respectively.

Thermogravimetric Analysis: In an air flow, the thermal stability of Yb(TTA)_2_AAPhen and Nd(TTA)_2_AAPhen as well as the copolymers were evaluated using thermogravimetric analysis (TGA, Netzsch SAT 449C, Selb, Germany) scanning from room temperature to 800 °C at the heating rate of 10 °C·min^−1^.

Thermal Properties Analysis: Differential scanning calorimetry (DSC, 204 Phoenix, Netzsch, Selb, Germany) was used to determine the thermal properties of the copolymers. The scanning temperature increased from room temperature to 150 °C, and decreased to −75 °C before increasing to 150 °C again. The heating and cooling were conducted under a nitrogen atmosphere at a rate of 10 °C·min^−1^.

X-ray diffraction: The structure characterization of Yb(TTA)_2_AAPhen and Nd(TTA)_2_AAPhen powders as well as the copolymers were performed using a Smartlab (Rigaku, Tokyo, Japn) thin-film diffractometer employing Cu K_α_ radiation (λ = 0.15046 nm), with the 2θ angle from 5° to 50° at the scanning rate of 10°·min^−1^.

Microscopy: The morphology of Yb(TTA)_2_AAPhen and Nd(TTA)_2_AAPhen powders as well as the copolymers were examined using a scanning electron microscope (SEM, JSM-6510, JOEL, Tokyo, Japan) equipped with a NORAN System 7 EDX detector (Thermo Fisher Scientific, Pittsburgh, PA, USA).

Photothermal Effect: The NIR lights of 980 and 808 nm were generated respectively by a laser diode driver (KS3-11312-912, BWT Co., Beijing, China) and a laser driver (FC-808-10W, Xinchanye Co., Changchun, China). The sample temperatures were measured using a hand-held infrared camera (Xintest Company, Dongguan, China). The power density was determined using an optical power/energy meter (Model 1918-R, Irvine, CA, USA) equipped with a thermopile detector (Model 818P-020-12, Newport).

Swelling Experiments: A piece of the sample with the mass of *m*_1_ was cut and immersed in DMF. After 72 h, the sample was removed from the solvent, while the mass (*m*_2_) was immediately measured in the swollen state after cleaning the extra solvent using a piece of tissue. The swollen sample was heated at 60 °C again until completely dry to obtain the final weight (*m*_3_). The gel content (*G*) was given by *m*_3_/*m*_1_ × 100%. The swelling degree (*Q*) was calculated using Equation (1). The *ρ*_1_ and *ρ*_2_ were the specific densities of the swelling solvent of DMF and PMMA.

(1) $$Q=\left[1+\rho_{2}\cdot \left(\frac{m_{2}}{m_{1}\cdot \rho_{1}}-\frac{1}{\rho_{1}}\right)\right]\times 100\%$$

The cross-linking density ([*XLD*]_s_ in moles per cubic centimeters) of the cross-linked copolymers was determined using the Flory-Rehner equation (Equation (2)) [46].

(2) $${\left[XLD\right]}_{s}=\frac{\ln\left(1-V_{R}\right)+V_{R}+\chi V_{R}^{2}}{2V_{s}\left(0.5V_{R}-V_{R}^{\frac{1}{3}}\right)}$$

where $V_{R}=\frac{m_{3}}{m_{3}+\left(m_{2}-m_{3}\right)\frac{\rho_{2}}{\rho_{1}}}$ was the volume fraction of epoxy network in the swollen sample. *V*_s_ was the molar volume of solvent, and χ was the solvent-polymer interaction parameter (0.50) [47].

Mechanical Properties: A flexural test instrument (MZ-2000c, Mingzhu Testing Machinery Co., Jiangdu, China) was used to perform the flexural test experiment via a three-point bending setup with a 30-mm span. The applied strain rate was 1.5 mm·s^−1^. The sample dimension was *L* × *W* × *D* = 60 mm × 15 mm × 0.9 mm. The flexural yield strength and flexural modulus were obtained based on the yielding point.

Shape-Memory Effect: The shape-memory effect was determined on the basis of angle variation. The test specimens were placed onto a hot plate at 150 °C. After 5 min, the specimens were completely folded to 90° against a perpendicular glass plate tightly and kept for another 5 min before gradual cooling to room temperature. Upon the irradiation of NIR light to the bended corner, the instantaneous angles, θ, between the real-time location and the primary location of the moving leg were characterized with the error of ±2°. The shape recovery ratio, *R*_r_, was determined as *R*_r_ = θ/90° × 100%.

## 3. Results and Discussion

### 3.1. Structure and Properties of Yb(TTA)_2_AAPhen and Nd(TTA)_2_AAPhen

**Figure 1:**
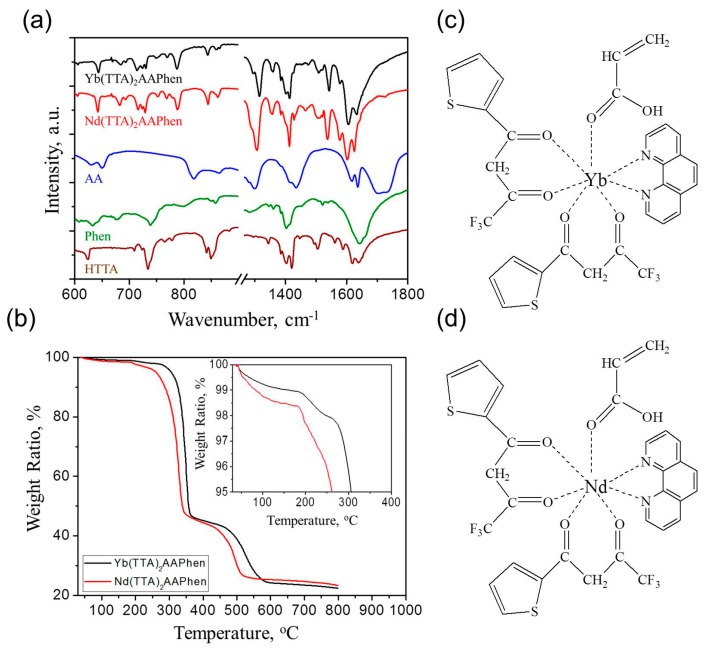
(**a**) FTIR spectra of AA, Phen, HTTA, Yb(TTA)_2_AAPhen and Nd(TTA)_2_AAPhen powders; (**b**) TGA curves in air flow of Yb(TTA)_2_AAPhen and Nd(TTA)_2_AAPhen powders; (**c**,**d**) Chemical structures of (**c**) Yb(TTA)_2_AAPhen and (**d**) Nd(TTA)_2_AAPhen.

To determine the chemical structures of Yb(TTA)_2_AAPhen and Nd(TTA)_2_AAPhen, FTIR spectra of the two RE complexes and the ligands are shown in Figure 1a. Three peaks related to the characteristic ring stretching vibration of Phen were located at 734, 852 and 1561 cm^−1^, which after coordination shifted to 713, 843 and 1542 cm^−1^ in Yb(TTA)_2_AAPhen and 716, 843 and 1537 cm^−1^ in Nd(TTA)_2_AAPhen, respectively [11,48]. The typical –C=C– (1618 cm^−1^) and –C=O– (1638 cm^−1^) stretching vibrations of AA varied to 1605 and 1633 cm^−1^ in Yb(TTA)_2_AAPhen as well as 1602 and 1625 cm^−1^ in Nd(TTA)_2_AAPhen [49]. The peaks at 1642 and 1663 cm^−1^, which were assigned to the –C=O– stretching vibration of HTTA, also contributed to the typical peaks in Nd(TTA)_2_AAPhen and Yb(TTA)_2_AAPhen [48]. It is worth mentioning that HTTA stands for α-thenoyltrifluoroacetone. During the formation of organic complexes, the hydrogen atom of the enol isomer of HTTA was off because of acid dissociation, generating TTA as the real ligand.

The TGA curves of Yb(TTA)_2_AAPhen and Nd(TTA)_2_AAPhen are illustrated in Figure 1b. The theoretical values of weight percentage of each component in Yb(TTA)_2_AAPhen and Nd(TTA)_2_AAPhen were calculated to be Yb: 19.5%, TTA: 50.1%, Phen: 22.3%, AA: 8.1% as well as Nd: 16.8%, TTA: 51.7%, Phen: 23.1%, AA: 8.4%. A slight weight loss initiating from 191 and 185 °C for Yb(TTA)_2_AAPhen and Nd(TTA)_2_AAPhen, respectively, was noticed, as shown in the insert figure, which was related to the decomposition of ligand AA in both samples [49]. Beginning from 270 and 247 °C, the second degradation attributing to the decomposition of ligand TTA took place, while the third degradation stepping from 464 and 441 °C resulted from the decomposition of ligand Phen. Similar to the reported Yb(TTA)_3_Phen and Nd(TTA)_3_Phen [11], the thermal resistance of Yb(TTA)_2_AAPhen was better than Nd(TTA)_2_AAPhen. The smaller atom radius may contribute to a stronger coordination connection. In addition, the weight losses within the last two degradation steps were close to 50% and 20%, respectively, which coincided with the mass fractions of TTA and Phen in Yb(TTA)_2_AAPhen and Nd(TTA)_2_AAPhen. The weight loss within the first degradation step was smaller than the theoretical weight ratio of AA because the following degradation of TTA resulted in an evident inflection point and retarded the measurements of its real ratio. The chemical structures of the two RE complexes, thus, are shown in Figure 1c,d on the basis of FTIR and TGA results.

**Figure 2:**
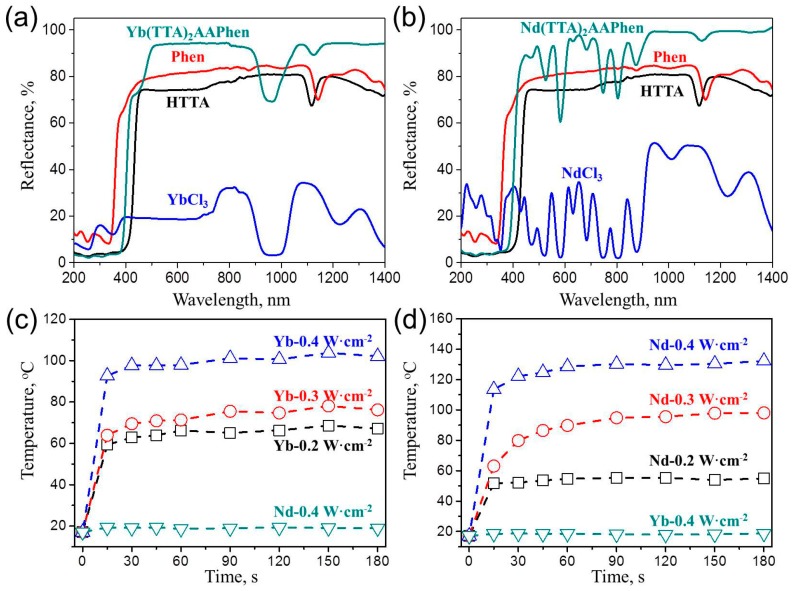
(**a**) Reflection spectra of Phen, HTTA, YbCl_3_ and Yb(TTA)_2_AAPhen; (**b**) Reflection spectra of Phen, HTTA, NdCl_3_ and Nd(TTA)_2_AAPhen; (**c**,**d**) The temperature increase of the powders with time upon the irradiations of (**c**) 980 and (**d**) 808 nm NIR light.

As a selectively responsive photothermal filler, the investigation on energy conversion route is of great importance. To demonstrate whether the complexes can absorb NIR lights with specific wavelengths, the reflection spectra of HTTA, Phen, YbCl_3_, NdCl_3_, Yb(TTA)_2_AAPhen and Nd(TTA)_2_AAPhen were detected as presented in Figure 2a,b. An absorption peak at 1126 nm was observed in both Yb(TTA)_2_AAPhen and Nd(TTA)_2_AAPhen, which superposed on the typical absorption peaks of 1119 and 1142 nm from HTTA and Phen, respectively, further indicating the successful coordination. More importantly, an obvious absorption peak of Yb(TTA)_2_AAPhen was located at 975 nm (Figure 2a), which can be related to the level transition from ^2^*I*_7/2_ to ^2^*F*_5/2_, while Nd(TTA)_2_AAPhen exhibited a clear peak at 804 nm (Figure 2b), which was contributed by the level transition from ^4^*I*_9/2_ to ^4^*F*_5/2_. More peaks were observed for Nd(TTA)_2_AAPhen due to its more energy levels. The peaks at 875, 749, 684, 629, 520, and 468 nm were respectively attributed to the lever transitions of ^4^*I*_9/2_ → ^4^*F*_3/2_, ^4^*I*_9/2_ → ^4^*F*_7/2_, ^4^*I*_9/2_ → ^4^*F*_9/2_, ^4^*I*_9/2_ → ^4^*H*_11/2_, ^4^*I*_9/2_ → ^4^*G*_5/2_, ^4^*I*_9/2_ → ^4^*G*_7/2_, and ^4^*I*_9/2_ → ^4^*G*_11/2_. No peaks around 808 and 980 nm were observed for Yb(TTA)_2_AAPhen and Nd(TTA)_2_AAPhen, indicating that Yb(TTA)_2_AAPhen and Nd(TTA)_2_AAPhen were discriminately sensitive to 980 and 808 nm. Therefore, as expected, Yb(TTA)_2_AAPhen and Nd(TTA)_2_AAPhen powders presented the selective photothermal effects upon the irradiations of 980 and 808 nm, respectively (Figure 2c,d), i.e., the temperatures of Yb(TTA)_2_AAPhen and Nd(TTA)_2_AAPhen varied upon different light wavelengths (980 and 808 nm) and power densities (0.2, 0.3 and 0.4 W·cm^−2^). Under the irradiation of 980 nm light with a power density of 0.2 W·cm^−2^, the temperature of Yb(TTA)_2_AAPhen increased from room temperature to 66 °C in 60 s, while increasing the power density to 0.3 and 0.4 W·cm^−2^ resulted in higher balanced temperatures at 76 and 102 °C (Figure 2c). Nd(TTA)_2_AAPhen, however, exhibited a neglected temperature change upon 980 nm light irradiation. On the other hand, Yb(TTA)_2_AAPhen showed no photothermal effect upon NIR light of 808 nm, while an obvious temperature increase to 55, 89, and 128 °C was observed for Nd(TTA)_2_AAPhen upon the irradiation of a certain NIR light with the power densities of 0.2, 0.3 and 0.4 W·cm^−2^ in 60 s (Figure 2d).

### 3.2. Structure and Properties of Light-Induced SMPs

**Scheme 2:**
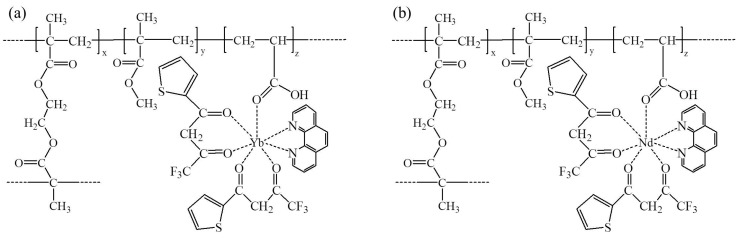
Schematic diagrams of (**a**) PMMA–Yb and (**b**) PMMA–Nd copolymers.

**Figure 3:**
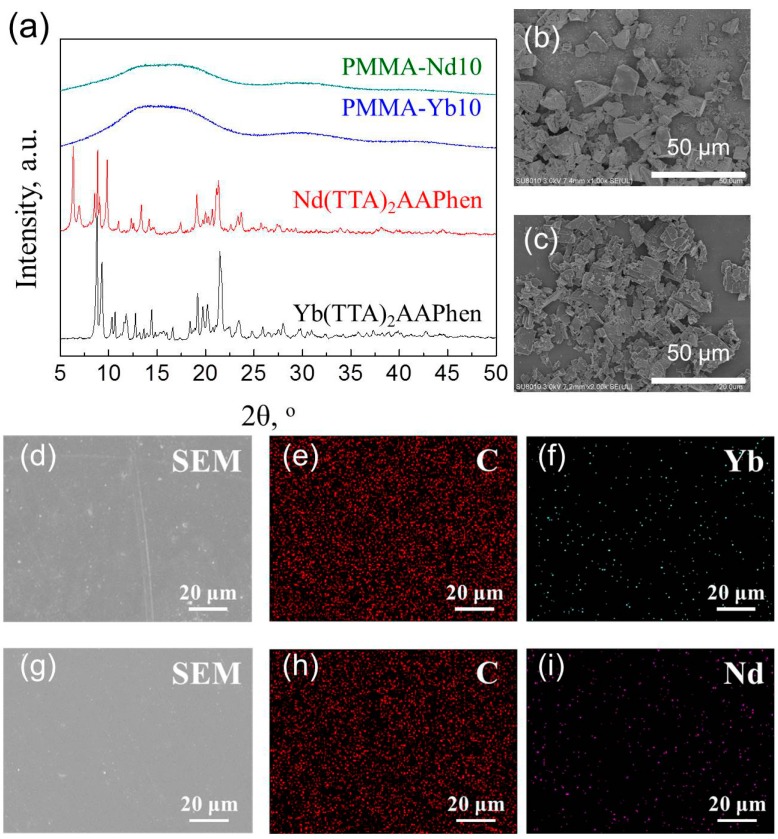
(**a**) XRD patterns of PMMA-Yb10, PMMA-Nd10, Yb(TTA)_2_AAPhen and Nd(TTA)_2_AAPhen; (**b**–**d**,**g**) SEM images of (**b**) Yb(TTA)_2_AAPhen, (**c**) Nd(TTA)_2_AAPhen, (**d**) PMMA–Yb10, and (**g**) PMMA–Nd10; (**e**,**f**,**h**,**i**) EDX mapping images of (**e**) C, and (**f**) Yb elements on Yb(TTA)_2_AAPhen as well as (**h**) C, and (**i**) Nd elements on Nd(TTA)_2_AAPhen.

The introduction of reactive double bonds through ligand AA into the prepared complexes was expected to enable a free radical copolymerization with MMA monomers and EGDMA as the cross-linker (Scheme 2). The filler dispersion was expected to be highly improved, which was verified using XRD characterization first (Figure 3a). The pristine complexes exhibited evident strong peaks and negligible amorphous scattering, indicating their highly crystalline structures. After in situ copolymerization, PMMA–Yb10 and PMMA–Nd10 both presented broad scatterings resulting from the amorphous PMMA matrix, while the typical crystal reflections generated by the complexes were absent, suggesting the disappearance of their primary crystalline structures. SEM and EDX were further conducted. As shown in Figure 3b,c, the pristine Yb(TTA)_2_AAPhen and Nd(TTA)_2_AAPhen presented powder-shaped and sheet-shaped structures with sizes up to ~20 μm. The in situ reaction as a chemical approach significantly improved their dispersions, i.e., almost no powders were observed from SEM images (Figure 3d,g). Besides, the Yb and Nd element signals dispersed uniformly as determined from the EDX mapping results, strongly demonstrating the homogenous existence of Yb(TTA)_2_AAPhen and Nd(TTA)_2_AAPhen in the copolymer matrix (Figure 3f,i).

**Figure 4:**
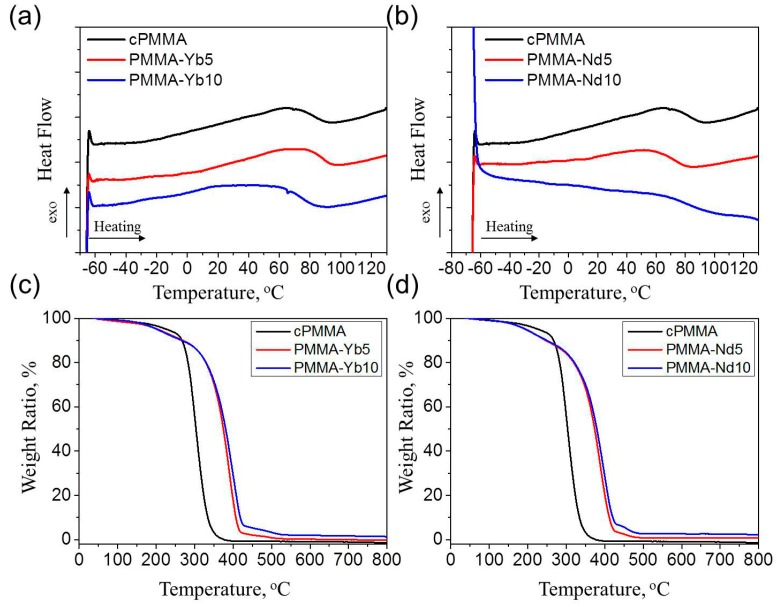
(**a**,**b**) The second heating DSC curves of cPMMA and copolymers; (**c**,**d**) TGA curves of (**c**) PMMA–Yb and (**d**) PMMA–Nd copolymers, in addition to cPMMA.

The effects of the dangling organic complexes on the thermal and mechanical properties of the prepared copolymers were investigated. As shown in Table 1, the flexural yield strength and flexural modulus of PMMA–Yb and PMMA–Nd decreased with complex loadings. The incorporation of 10 phr Yb(TTA)_2_AAPhen resulted in the reductions of 26% and 24% in flexural yield strength and flexural modulus, while Nd(TTA)_2_AAPhen of 10 phr contributed to reductions of 35% and 38% as well. DSC results, as shown in Figure 4a,b, were used to determine the *T*_g_ of the prepared copolymers. The introduction of organic complexes caused a variation of ~10 °C in their *T*_g_s. More specifically, the overall tendency was that the copolymers possessed lower *T*_g_s than the pristine cPMMA. The gel content (*G*) and swelling ratio (*Q*) of cPMMA as well as SMPs containing different amounts of RE organic complexes were measured (Table 1). The dense network was achieved in cPMMA as determined by its high *G* of 99.3% and low *Q* of 169%. The in situ reaction with Yb(TTA)_2_AAPhen and Nd(TTA)_2_AAPhen reduced the cross-linking level, i.e., the values of *G* and *Q* gradually reduced and increased, respectively. It should be noted that a *G* over 90% demonstrated the generation of enough netpoints in a polymer matrix after in situ reaction [50]. The [*XLD*]_s_ was also calculated based on the swelling experiment, while the data clearly showed a reduction in cross-linking density (Table 1). It was expected that the steric hindrance resulting from the organic complex should increase the chain stiffness and as a result the modulus and *T*_g_. However, the large dangling group also retarded the copolymerization of EGDBA as the cross-linker and resultantly facilitated the formation of a loose network. The decrease in cross-linking density provided the prepared copolymers good chain mobility than the pristine cPMMA, reducing the modulus and *T*_g_.

As illustrated by the TGA curves in Figure 4c,d, the degradation initiated earlier for the copolymers containing complexes possibly due to AA decomposition, i.e., the temperature at the 5% weight loss decreased from 227 °C for cPMMA to 206, 202, 194 and 195 °C for PMMA–Yb5, PMMA–Yb10, PMMA–Nd5, and PMMA–Nd10, respectively. Upon increasing the temperature further, the thermal resistance of the copolymers was evidently improved with the aid of organic complexes to increase chain stiffness. A great increase of 70–80 °C in the temperature at the 50% weight loss was observed, while the temperatures at the 90% weight loss of the copolymers were 65–86 °C higher than cPMMA. Besides, thermal resistance was positively correlated to complex loadings.

**Figure 5:**
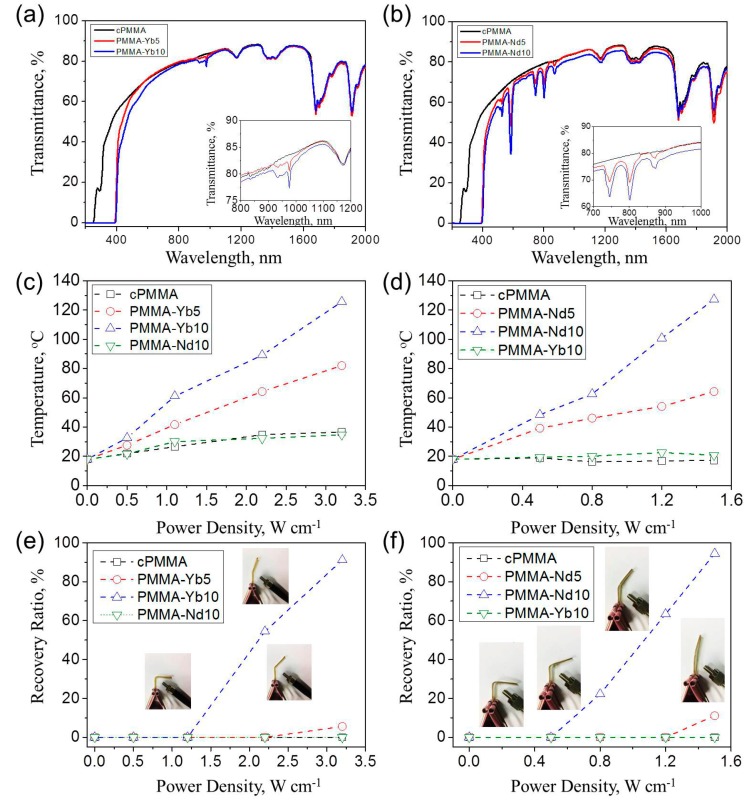
(**a**,**b**) Transmittance spectra of (**a**) PMMA–Yb and (**b**) PMMA–Nd samples with the thickness of ~1 mm; (**c**,**d**) Temperature vs. power density curves of PMMA copolymers upon the irradiation of (**c**) 980 and (**d**) 808 nm; (**e**,**f**) Recovery ratio of PMMA copolymers upon the irradiation of (**e**) 980 and (**f**) 808 nm.

The discriminated absorptivity of the synthesized polymers was investigated using UVPC to ensure that the in situ reaction did not influence the certain absorption peaks as behaved by the complexes themselves (Figure 5a,b). In comparison with air as the reference, the cPMMA specimen with the thickness of 1.0 ± 0.1 mm presented good transmittance between 60% and 80% in the visible light range (400–800 nm). Because of the good dispersion resulting from the in situ reaction, the dangling organic complexes varied the transmittance negligibly, while the ligands of TTA and Phen highly increased the absorption in the UV light range (200–400 nm). More importantly, Yb(TTA)_2_AAPhen and Nd(TTA)_2_AAPhen, as expected, generated new peaks located at 975 and 802 nm, respectively, compared to pristine cPMMA. Higher loading increased the peak intensity, i.e., the transmittance at 975 nm decreased from 83.6% for cPMMA to 80.6% and 77.3% for PMMA–Yb5, and PMMA–Yb10, respectively. At the wavelength of 802 nm, cPMMA, PMMA–Nd5, and PMMA–Nd10 exhibited a gradually decreased transmittance from 79.2 to 69.2% and 62.7% as well. Due to such certain absorption peaks, the prepared polymers also presented selective photothermal effects upon the NIR light irradiation of 980 or 808 nm, as shown in Figure 5c,d. The NIR light of 980 nm was unable to cause the temperature raise of cPMMA and PMMA–Nd10, while PMMA–Yb5 and PMMA–Yb10 were heated in a linear manner with the power density, i.e., their temperatures increased from room temperature to 82 and 126 °C at the power density of 3.2 W·cm^−1^ (Figure 5c). Similarly, the NIR light of 808 nm did not increase the temperature of cPMMA and PMMA–Yb10 because of the absent photothermal filler. PMMA–Nd5 and PMMA–Nd10, however, can increase their temperatures at 1.5 W·cm^−1^ to 64 and 127 °C (Figure 5d).

The light-induced shape recovery ratio (*R*_r_) highly relied on the suitable light wavelength and power density, which dominated the indirectly generated temperature (Figure 5e,f). The PMMA–Nd10 and PMMA–Yb10 did not initiate their deformations at 980 and 808 nm, and cPMMA did not present shape recovery either. Good shape recovery (91% and 94%) occurred only for PMMA-Yb10 and PMMA–Nd10 when the power densities of the NIR lights of 980 and 808 nm went beyond 3.2 and 1.5 W·cm^−2^, where the measured temperatures were higher than their *T*_g_s. Quantitative results and the qualitative photos, as shown in Figure 5e,f, indicated that the in situ copolymerization of Yb(TTA)_2_AAPhen and Nd(TTA)_2_AAPhen enabled the SMPs to recover from temporary shape upon selective NIR light irradiations. It is worth mentioning that since the temperature increase not only depended on the filler content but the light intensity as well, increasing the light power density was expected to trigger the shape recovery of PMMA–Yb5 and PMMA–Nd5 ultimately, which was not further investigated.

### 3.3. Applications in Smart Optical Devices

In addition to the macroscale shape deformation under the exposure towards certain NIR lights, the recoveries of the microstructures on PMMA–Yb10 and PMMA–Nd10 surfaces were also explored. The good transparency resulted from the fact that in situ copolymerization highly improved the dispersion of RE organic complexes with a high loading of 10 phr in the polymer matrix offered the opportunity to create smart optical devices. It is difficult for other conventional photothermal fillers to achieve such neglected influence on the transparency.

**Figure 6:**
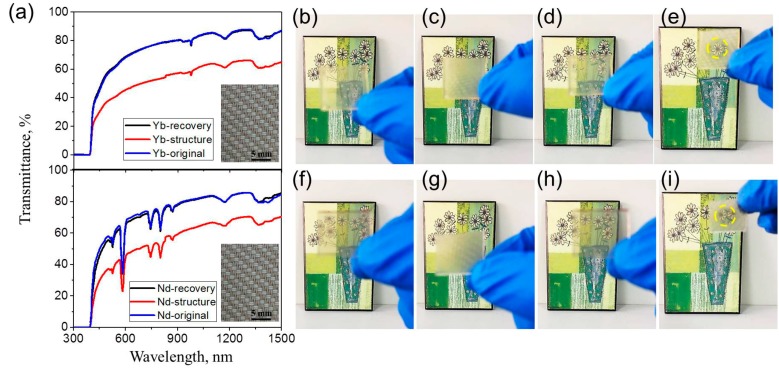
(**a**) Transmittance curves of PMMA-Yb10 and PMMA–Nd10 with the original, temporary, and recovered surface topographies. The inserted image presents the topography of the used Nylon fabric. (**b**–**i**) Photos of (**b**–**e**) PMMA-Yb10 and (**f**–**i**) PMMA–Nd10 with the (**b**,**f**) original, (**c**,**g**) temporary, (**d**,**h**) recovered, and (**e**,**i**) locally recovered surface topography.

Two pieces of Nylon fabrics (shown in the inserted image in Figure 6a) were first compressed onto the two sides of PMMA–Yb10 or PMMA–Nd10 at a high temperature of 150 °C, which was over their *T*_g_ (see Scheme 1). Because of the appearance of the temporary rough surfaces, the transmittance of PMMA–Yb10 decreased from 69.0 to 45.7% at the wavelength of 600 nm for an instance (Figure 6a), while the sample became opaque (Figure 6c) in comparison with the original status (Figure 6b). Heating up to 150 °C triggered the complete recovery of the surface topography and the sample transparency (Figure 6d). The transmittance at the wavelength of 600 nm also increased to 68.4%, suggesting a recovery ratio of 99% (Figure 6a). Here, upon the precise irradiation of 980 nm NIR light onto the on-demand area of the compressively deformed PMMA–Yb10, a localized shape recovery was achieved as indicated by the yellow circle and the scene behind appeared (Figure 6e). Similar selective microstructure recovery was also observed for PMMA–Nd10 upon the irradiation of 808 nm NIR light (Figure 6f–i). As reported by a previous research, a 3 × 3 array of ITO heaters was used to realize the sequential recovery of different regions of an EVA film with a compressively deformed microprism array [40]. By contrast, the usage of a selectively photo-responsive SMP obviously achieved the remote and precise control of the shape recovery at randomly localized areas.

**Figure 7:**
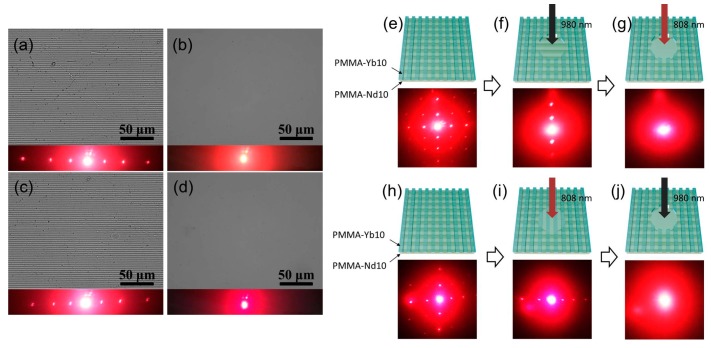
(**a**–**d**) OM images and diffraction patterns of (**a**,**b**) PMMA–Yb10 and (**c**,**d**) PMMA–Nd10 with the (**a**,**c**) temporary gratings and (**b**,**d**) recovered surfaces. (**e**–**j**) Schematic diagrams and diffraction patterns of the cross-stacked PMMA–Yb10 (front) and PMMA–Nd (back) with temporary grating structures upon different irradiations of 980 or 808 nm NIR light.

Smart soft grating devices have also been reported, resulting from a deformation of surface structures at the micro-/nanoscale [42,51]. Further, instead of fabricating the temporary opaque sample, a glass grating with the grating parameter of 3.3 μm was used to create grating structures at the microscale on PMMA–Yb10 and PMMA–Nd10, for the purpose of fabricating light-induced soft grating devices. As shown in Figure 7a–d, the temporary grating structures on PMMA–Yb10 (Figure 7a) or PMMA–Nd10 (Figure 7c) disappeared upon 150 °C, while the diffraction patterns also vanished, generating round halos (Figure 7b,d). Such transparent soft grating materials with the capability of selectively photo-responsive shape-memory effect can be assembled to prepare smart grating devices. As shown in Figure 7e,h, after the temporary grating microstructures were created on PMMA–Yb10 and PMMA–Nd10 separately, the two specimens were layered together at a 90 degree angle. A lattice diffraction pattern, thus, was achieved. Upon irradiation at 980 nm, the grating structures on the front PMMA–Yb10 layer disappeared, while the PMMA–Nd10 layer on the back were not affected, diffracting the light into a perpendicular diffraction strip (Figure 7f). The subsequent irradiation of 808 nm penetrated the front PMMA–Yb10 layer and resulted in the complete vanishing of the grating structures on PMMA–Nd10 and the appearance of a halo pattern (Figure 7g). Switching the irradiation order varied the evolution procedure of the diffraction patterns. The NIR light of 808 nm, which did not trigger the shape recovery of the front PMMA–Yb10, caused the disappearance of the grating structures on PMMA–Nd10, while the parallel diffraction strip was achieved (Figure 7i). The 980 nm light finally led to the halo pattern due to the shape recovery on the front PMMA–Yb10 (Figure 7j).

## 4. Conclusions

Via free radical copolymerization, shape-memory polymers selectively responsive to NIR lights of 980 or 808 nm were synthesized. The usage of acrylic acid containing double bonds offered the prepared Yb(TTA)_2_AAPhen and Nd(TTA)_2_AAPhen reactivity with methyl methacrylate monomers and ethylene glycol dimethylacrylate. Increasing the contents of organic complexes enhanced the thermal resistance of the copolymers, but caused a reduction in glass transition temperature, cross-link density, and mechanical properties. The transparency, however, was not affected evidently. Further, the discriminated absorptivity of the complexes close to the wavelengths of 980 and 808 nm provided the copolymers with the selective photothermal effect and shape-memory capability. Finally, smart optical devices were created which can switch the localized transparency or the diffraction evolution procedures. Further work should focus on the development of other organic complexes containing different reactive ligands and the in situ synthesis of other NIR light-induced SMPs.

## Figures and Tables

**Table 1:** Composition, mechanical, thermal properties of PMMA-copolymers.

Sample name	σ ^a^	*E* ^b^	*T*_g_^c^	*G* ^d^	*Q* ^e^	*[XLD]_s_^f^*	*T*_d,5_^g^	*T*_d,50_^h^	*T*_d,90_^i^
	MPa	GPa	°C	%	%	mmol cm^−3^	°C	°C	°C
cPMMA	45.5 ± 2.4	2.1 ± 0.1	86	99.3 ± 0.2%	169 ± 3%	0.62 ± 0.06	227	304	335
PMMA-Yb5	41.4 ± 2.9	1.9 ± 0.2	87	94.7 ± 0.8%	198 ± 13%	0.26 ± 0.07	206	374	400
PMMA-Yb10	33.5 ± 5.2	1.6 ± 0.2	78	92.5 ± 1.2%	207 ± 2%	0.19 ± 0.02	202	380	418
PMMA-Nd5	35.3 ± 3.9	1.6 ± 0.2	73	94.9 ± 0.5%	202 ± 5%	0.23 ± 0.02	194	374	412
PMMA-Nd10	29.4 ± 2.1	1.3 ± 0.2	82	93.1 ± 0.6%	213 ± 23%	0.19 ± 0.10	195	379	421

^a, b^: Flexural yield strength (σ) and flexural modulus (*E*) measured from the three-point bending test at room temperature. ^c^: Glass transition temperature determined from DSC characterization; ^d, e^: Gel content (*G*) and swelling ratio (*Q*) obtained from swelling experiments; ^f^: Cross-linking density [*XLD*]_s_ calculated from swelling experiments; ^g–i^: Temperatures at 5%, 50%, and 90% weight loss, determined from TGA curves.
